# Caveolin‐1 negatively regulates inflammation and fibrosis in silicosis

**DOI:** 10.1111/jcmm.17045

**Published:** 2021-12-09

**Authors:** RongLing He, XiangNing Yuan, Xin Lv, QingXiang Liu, LiJian Tao, Jie Meng

**Affiliations:** ^1^ Department of Pulmonary and Critical Care Medicine Third Xiangya Hospital Central South University Changsha China; ^2^ Hunan Key Laboratory of Organ Fibrosis Central South University Changsha China; ^3^ Department of Nephrology Xiangya Hospital Central South University Changsha China

**Keywords:** caveolin‐1, fibrosis, inflammation, silicosis

## Abstract

Inhalation of crystalline silica causes silicosis, the most common and serious occupational disease, which is characterized by progressive lung inflammation and fibrosis. Recent studies revealed the anti‐inflammatory and anti‐fibrosis role of Caveolin‐1 (Cav‐1) in lung, but this role in silicosis has not been investigated. Thus, this study evaluated Cav‐1 regulatory effects in silicosis. It was found that Cav‐1 levels were significantly reduced in the lung from silicosis patients and silicotic mice. The silicosis models were established in C57BL/6 (wild‐type) and Cav‐1 deficiency (*Cav*‐*1* ^−/−^) mice, and *Cav*‐*1* ^−/−^ mice displayed wider alveolar septa, increased collagen deposition and more silicotic nodules. The mice peritoneal‐derived macrophages were used to explore the role of Cav‐1 in silica‐induced inflammation, which plays a central role in mechanism of silicosis. Cav‐1 inhibited silica‐induced infiltration of inflammatory cells and secretion of inflammatory factors in vitro and in vivo, partly by downregulating NF‐κB pathway. Additionally, silica uptake and expression of 4‐hydroxynonenal in silicotic mice were observed, and it was found that Cav‐1 absence triggered excessive silica deposition, causing a stronger oxidative stress response. These findings demonstrate the protective effects of Cav‐1 in silica‐induced lung injury, suggesting its potential therapeutic value in silicosis.

## 1 INTRODUCTION

Silicosis caused by long‐term exposure to dust containing free crystalline silica is the most common and serious occupational disease in China and other developing countries.^1^ The lung cannot expel insoluble silica, which continuously stimulates macrophages and cause gradual and cumulative damage to the pulmonary parenchyma, resulting in diffuse chronic inflammation, formation of silicotic nodules and progressive fibrosis.^2^ Patients with silicosis have progressive clinical lung function decline even 20 years after leaving the dust environment,^3^ and eventually, suffer respiratory failure. Silicosis has no specific treatment except lung transplantation.^4^ Therefore, knowledge of the pathogenesis and effective therapeutic targets of silicosis is critical.

Caveolae are invaginations in the plasma membrane of cells that regulate signal transduction, lipid metabolism and receptor‐mediated endocytosis.^5^ Caveolin‐1 (Cav‐1) is the caveolae major functional protein, highly expressed in lungs.^6^ Cav‐1 regulates lung fibrosis and inflammatory response. In lungs from bleomycin‐induced mice and radiation‐induced rats, Cav‐1 deficiency aggravated fibroblast proliferation and collagen deposition.^7^, ^8^ Cav‐1 inhibited the infiltration of inflammatory cells and the secretion of inflammatory factors in lungs from lipopolysaccharide (LPS)‐instilled mice.^9^, ^10^ Furthermore, Cav‐1 inhibited macrophages activation through multiple pathways.^10^, ^11^

The role of Cav‐1 in silicosis has not been investigated. Therefore, this study aimed to elucidate the function of Cav‐1 in silicosis. The results showed that Cav‐1 protected against lung injury in silicosis. Cav‐1 also inhibited silica‐induced inflammation, partly by regulating the NF‐κB pathway, silica intake and oxidative stress. These results provide new ideas for exploring the silicosis mechanism and propose that Cav‐1 is a potential silicosis therapeutic target.

## 2 MATERIALS AND METHODS

### 2.1 Subjects

Paraffin‐embedded sections of biopsy specimens were collected from nine silicosis subjects. A local pneumoconiosis diagnosis group diagnosed silicosis following the GBZ25‐2014 standard issued in China. None of the silicosis subjects presented clinical signs of autoimmune diseases, active stage tuberculosis or lung tumour. Paraffin‐embedded sections of normal tumour‐adjacent tissues from nine lobectomy specimens were collected as the control group. None of the control subjects had a history of occupational exposure history to silica dust. The Medical Ethics Committee of Third Xiangya Hospital of Centre South University approved this study (permit number: 2020‐S269).

### 2.2 Animals

Male C57BL/6 mice were purchased from Silaike Laboratory. The Yuan Lei group from Shanghai Medical College of Fudan University provided Cav‐1 knockout (*Cav*‐*1* ^−/−^) mice free of charge.^12^ All animal experiments were conducted following the Animal Care and Use Committee of Central South University guidelines (permit number: 2020sydw0474).

### 2.3 Establishment of the silicosis mouse model

There were two animal experiments: (1) Cav‐1 expression in the lungs of silicotic mice; (2) the effects of Cav‐1 deficiency in silicotic mice. In the first experiment, male C57BL/6 mice (8 weeks old, 22–24 g) were divided into three groups as follows: (1) sham group, (2) silica 7 day group, (3) silica 28 day group. In the second experiment, C57BL/6 mice (wild‐type [WT]) and *Cav*‐*1* ^−/−^ mice (8 weeks old, 20–24 g) were divided into four groups as follows: (1) WT sham group, (2) WT silica 28 day group, (3) *Cav*‐*1* ^−/−^ sham group and (4) *Cav*‐*1* ^−/−^ silica 28 day group. There were five mice in each group.

On day 0, the silicosis model mice were intratracheally injected with 25 μl silica (U.S Silica Company; MIN‐U‐SIL5) suspension (200 mg/ml, equal to 5 mg/mouse).^13^ The mice in the sham group were intratracheally injected with 25 μl sterile saline, as previously described.^14^

In the first experiment, all mice were euthanized 28 days after treatment, except mice in the silica 7 day group, which were euthanized 7 days after treatment. In the second experiment, all mice were euthanized 28 days after treatment.

### 2.4 Bronchoalveolar lavage fluid isolation

Bronchoalveolar lavage fluid (BALF) was collected from euthanized mice at designated time points. BALF samples were centrifuged at 1000 *g* for 10 min at 4℃. The protein concentrations of cell‐free supernatants were measured with a bicinchoninic acid protein assay kit (Thermo Fisher Scientific). The cell pellets were re‐suspended in 1 ml phosphate‐buffered saline for cell counting using Countess 3 Automated Cell Counters (Invitrogen).

### 2.5 Lung tissues collection

Left lungs were collected and fixed with 4% paraformaldehyde for histopathological analysis. Right lungs were preserved at 80℃ for Western blot (WB) and Reverse transcription real‐time polymerase chain reaction (RT‐qPCR) analyses.

### 2.6 Lung histology

Fixed lungs were dehydrated, embedded in paraffin and cut into 4 μm‐thick slices. The lung tissue sections were stained with haematoxylin‐eosin (HE) and Masson trichrome staining. Histological examination and photomicrography were conducted using a scanner (3DHISTECH).

Pulmonary fibrosis was quantified as eight grades according to the Ashcroft score as previously described.^15^ Silicotic nodules were graded as follows: cellular nodules as Stage I, scores 1‐point; fibrotic cellular nodules as Stage II, scores 1.2‐points; cellular fibrotic nodules as Stage III, scores 1.4‐points; and fibrotic nodules as Stage IV, scores 1.6‐point.^16^ The total silicotic nodules of each sample and the total number of points were counted and calculated, respectively.

### 2.7 Immunohistochemistry

The mice lung sections were evaluated for immunohistochemical localization of Cav‐1 (CST; #3267, 1:800), collagen I (Abcam; ab34710, 1:400), CD68 (Boster; BA3638, 1:200), myeloperoxidase (MPO) (Servicebio; G1311224, 1:400) and 4‐hydroxynonenal (4‐HNE) (Bioss; bs‐6313R, 1:200). The human lung sections were evaluated for immunohistochemical localization of Cav‐1 (CST; #3267, 1:800). The positive staining area was calculated from five random and noncoincident fields in each section at ×200 using the Image‐Pro‐Plus software to quantify expression of caveolin‐1, collagen I and 4‐HNE. Neutrophil (MPO) and macrophage (CD68^+^ cell) quantification were determined in 10 random and noncoincident fields in each section at ×100.

### 2.8 Warthin‐Starry silver staining of the silica

The histological sections were stained with 1% silver nitrate in a 43℃‐water bath for 30 min. After washing, the enzyme reaction was developed with a colour liquid containing 1.5 ml of 2% silver nitrate, 2 ml of 0.15% hydroquinone and 3.75 ml of 5% gelatin solution (1% citric acid solution, pH 4). When the sections turned brown‐yellow, the reaction was terminated using preheated water (54℃). Finally, they were dehydrated and mounted.

### 2.9 Isolation and culture of peritoneal‐derived macrophages

Peritoneal‐derived macrophages (PDMs) are commonly used for research in various diseases^17^ and simulate the inflammation of alveolar macrophages in silicosis.^18^ Therefore, this study employed silica‐stimulated PDMs to explore the Cav‐1 mechanism in silicosis.

The PDMs were isolated from WT and *Cav*‐*1* ^−/−^ mice as previously described.^19^ After 12 h of serum starvation, cells were exposed to 50 μg/ml silica (Nano‐SiO_2_; Invivogen).^20^, ^21^ The cells were harvested for protein extraction after treatment for 3 and 24 h, respectively. Supernatants of 24 h‐treated cells were centrifuged at 1800 *g* for 5 min at 4℃. After that, the cell‐free supernatants were preserved at −80℃ for cytokine detection.

In the caveolin‐1 overexpression experiment, PDMs were transiently transfected with pcDNA3.1(+)‐caveolin‐1 (Cav‐1 OE group) and pcDNA3.1(+) (Vehicle group) purchased from Genepharma Technology. Cells were prepared in 12‐well plates. When the cells reached 70–80% confluence, plasmids (1 µg per well) were introduced using 2 μl Lipofectamine max (Invitrogen) following the manufacturer's instructions. After 6 h of incubation, cells were cultured in RPMI 1640 medium supplemented with 10% fetal bovine serum for 48 h, followed by 50 μg/ml silica exposure for 3 and 24h, respectively.

### 2.10 Western blot

Proteins were extracted from lung tissues and cells. Target proteins were separated on 8%–12% sodium dodecyl sulphate‐polyacrylamide gels (according to the target protein sizes: 8% for collagen I, 10% for proteins in NF‐κB pathway, 12% for Cav‐1) and subsequently transferred onto polyvinylidene difluoride membranes. The membranes were blocked for 1 h in 5% bovine serum albumin (BSA) buffer and incubated with primary antibodies in 5% BSA at 4℃ overnight. The primary antibodies were as follows: rabbit anti‐caveolin‐1 (#3267, 1:5000), rabbit anti‐p‐NF‐κB(#3033, 1:1000), rabbit anti‐p‐IκBα (#2859, 1:1000), rabbit anti‐NF‐κB (#8242, 1:1000) and rabbit anti‐IκBα (#9242, 1:1000) from Cell Signaling Technology; rabbit anti‐collagen I (ab34710, 1:1000) from Abcam; mouse anti‐GAPDH (60004‐1‐Ig, 1:10,000), rabbit anti‐α‐tubulin (11224‐1‐AP, 1:5000) and mouse anti‐β‐actin (66009‐1‐Ig,1:20000) from ProteinTech. The membranes were incubated with horseradish peroxidase‐conjugated anti‐rabbit and anti‐rat for 60 min for detection. Protein expression was normalized using α‐tubulin and GAPDH or β‐actin.

### 2.11 Reverse transcription real‐time polymerase chain reaction (RT‐qPCR)

Total RNA was isolated from the lung tissues using TRIzol (Invitrogen) following the manufacturer's instructions. A Revert Aid First Strand cDNA Synthesis Kit reverse‐transcribed the RNA into DNA (Invitrogen). RT‐qPCR was performed using a CFX96 Quantitative PCR Detection System (Bio‐Rad). Specific primers were designed from GenBank sequences and synthesized by Sangon Biotech.

### 2.12 Enzyme linked immunosorbent assay

The interleukin (IL)‐1β, IL‐6 and tumour necrosis factor‐α (TNF‐α) levels in the supernatants were detected by enzyme linked immunosorbent assay (ELISA), following the manufacturer's instructions (Invitrogen).

### 2.13 Statistical analysis

The SPSS 25 statistical software was used to analyse the data. All data were expressed as the mean, standard deviation. The two‐tailed Student *t* test analysed comparisons between two groups. *p* < 0.05 was considered statistically significant.

## 3 RESULTS

### 3.1 Caveolin‐1 was significantly reduced in lungs from silicosis patients and silicotic mice

**FIGURE 1:**
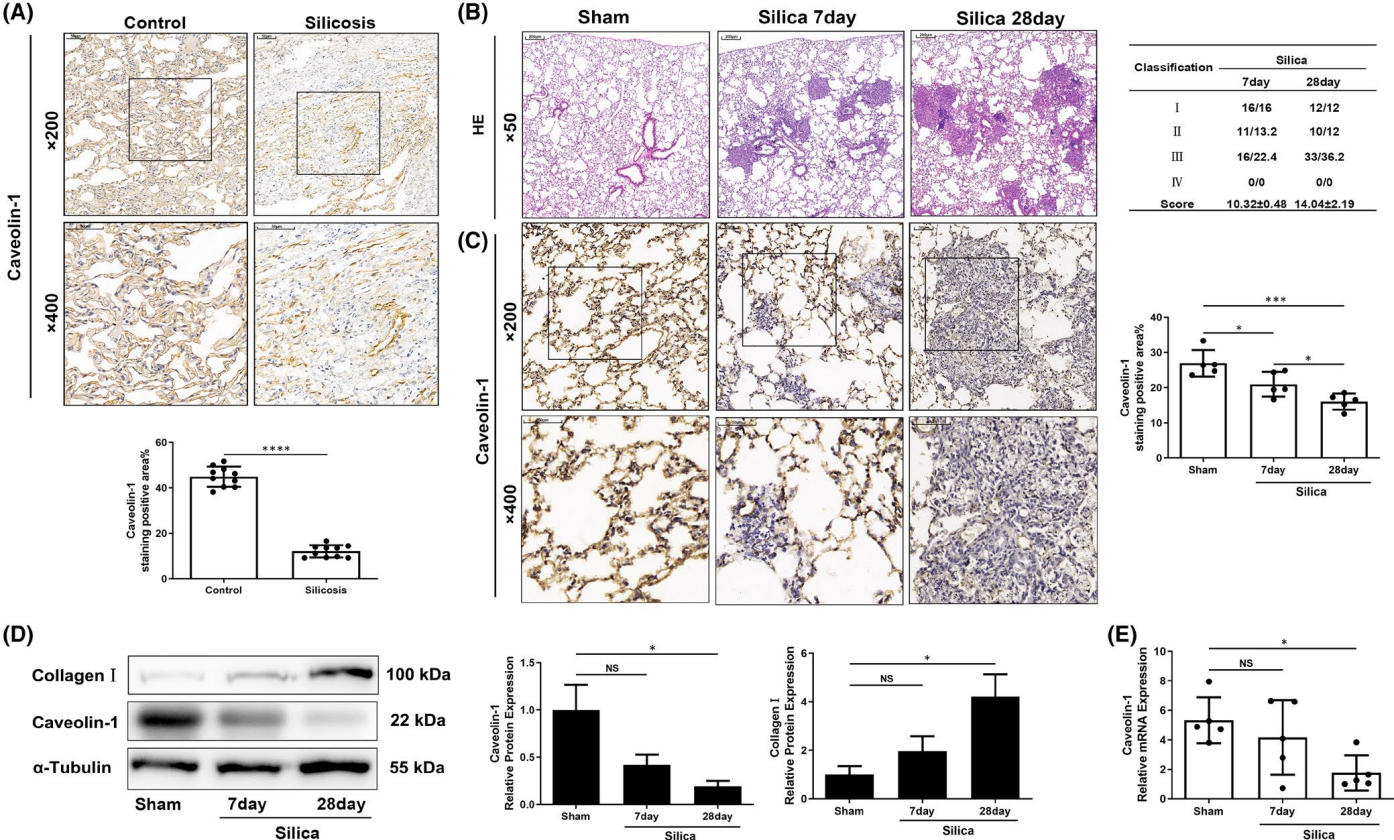
Caveolin‐1 (Cav‐1) was significantly reduced in lungs of silicosis patients and silicotic mice. (A) Immunochemistry staining of caveolin‐1 of lung sections from silicosis patients and normal tumour‐adjacent tissues (*n* = 9). (B) Haematoxylin‐eosin (HE) staining of lung sections from silicotic mice sampled at 7 and 28 days (*n* = 5), and silicotic nodules score in the silicosis group. (C) Immunochemistry staining of caveolin‐1 in the lung sections from silicotic mice sampled at 7 and 28 days (*n* = 5). (D) Western blot analysis of caveolin‐1 and collagen Ⅰ in lung tissues from silicotic mice sampled at 7 and 28 days (*n* = 3). (E) Caveolin‐1 relative mRNA levels in lung tissues from silicotic mice sampled at 7 and 28 days (*n* = 5). *p < 0.05, ***p < 0.001, ****p < 0.0001, NS: no significance

Paraffin‐embedded sections of lung tissues from nine patients with silicosis and nine cases of tumour‐adjacent tissues used as control were collected, and immunohistochemistry (IHC) was performed to measure the Cav‐1 expression. The IHC staining showed that the Cav‐1 level was high in normal tumour‐adjacent tissues but significantly decreased in silicosis lung tissues (*p* < 0.0001) (Figure 1A).

The silicosis model was established in WT mice for 7 and 28 days to detect the Cav‐1 level in silicosis. The lung tissue damage, silicotic nodule formation and inflammation infiltration progressed with increasing modelling time (Figure 1B). In mice from the silica 7 day group, part of the alveolar wall was destroyed, causing alveolar fusion. Around the small airway, silicotic nodules and inflammatory cells were observed. In mice from the silica 28 day group, part of the alveolar wall was remodelled into a continuous fibre wall with collagen deposition. The size and amount of silicotic nodules increased, and inflammatory cells diffusely infiltrated (Figure 1B, Table 1). The formation of silicotic nodules confirmed the silicosis model.

**TABLE 1:** Nucleotide sequences of the primers used for real‐time qPCR

Genes	Forward primer 5′−3′	Reverse primer 5′−3′
Mouse TNF‐α	CACCACGCTCTTCTGTCTACT	AACTGATGAGAGGGAGGCCAT
Mouse IL‐1β	CTGGTGTGTGACGTTCCCAT	TCGTTGCTTGGTTCTCCTTGT
Mouse IL‐6	ACCAAGAGATAAGCTGGAGTCAC	TAACGCACTAGGTTTGCCGA
Mouse β‐actin	CACTGTCGAGTCGCGTCC	TCATCCATGGCGAACTGGTG

Abbreviations: IL, interleukin; TNF‐α, tumour necrosis factor‐α.

Immunohistochemistry, WB and RT‐PCR determined the Cav‐1 expression (Figure 1C–E). The Cav‐1 protein levels time‐dependently reduced in the lungs of silicotic mice after different time points of silica exposure. However, the transcription‐level analysis showed no significant difference in the Cav‐1 reduction in the silica 7 day group compared with the control group. Nevertheless, the Cav‐1 in the silica 28 day group significantly decreased against the control group (*p* < 0.05).

### 3.2 Caveolin‐1 deficiency exacerbated silicotic lung injury and fibrosis

**FIGURE 2:**
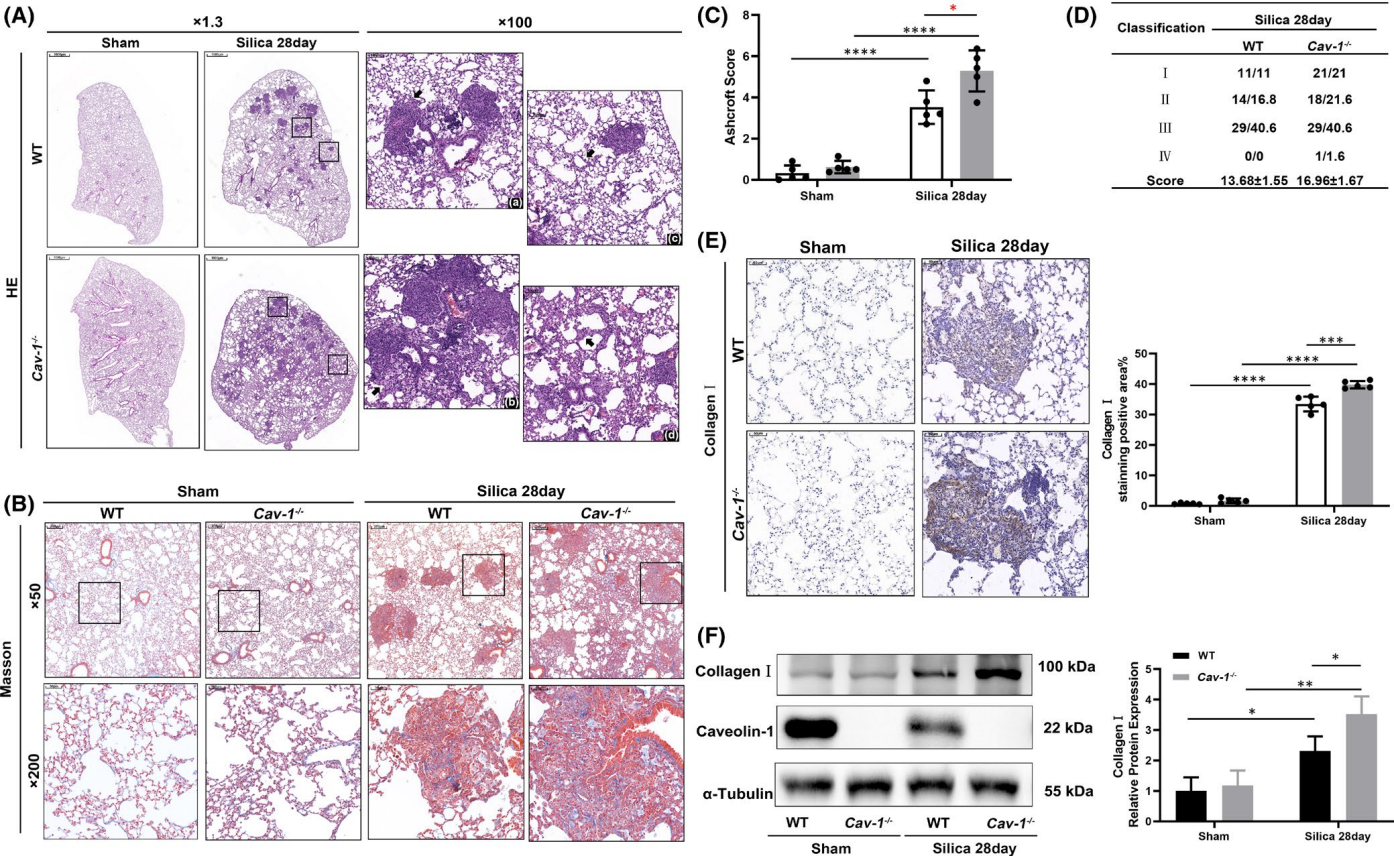
Caveolin‐1 (Cav‐1) deficiency exacerbated silicotic lung injury and fibrosis. (A) Haematoxylin‐eosin (HE) staining of lung sections from wild‐type (WT) and *Cav*‐*1* ^−/−^ mice in the sham and silica 28 day groups. (a) The WT group showed isolated silicotic nodules. (b) The *Cav*‐*1* ^−/−^ group showed fused silicotic nodules accompanied by more I and II‐degree nodules. (c, d) The *Cav*‐*1* ^−/−^ group showed more cells and fibre deposition in the alveolar septum than the WT group. (B) Masson staining of lung sections from WT and *Cav*‐*1* ^−/−^ mice in the sham and silica 28 day groups. (C) Ashcroft score of WT and *Cav*‐*1* ^−/−^ mice in the sham and silica 28 day groups (*n* = 5). (D) Silicotic nodules score of WT and *Cav*‐*1* ^−/−^ mice in the sham and silica 28 day groups (*n* = 5). (E) Collagen Ⅰ immunochemistry staining of lung sections from WT and *Cav*‐*1* ^−/−^ mice in the sham and silica 28 day groups (*n* = 5). (F) Western blot analysis of Cav‐1 and collagen Ⅰ in lung tissues from WT and *Cav*‐*1* ^−/−^ mice in the sham and silica 28 day groups (*n* = 3). *p < 0.05, **p < 0.01, ***p < 0.001, ****p < 0.0001

The silicosis model was established in WT and *Cav*‐*1* ^−/−^ mice for 28 day. Based on HE staining, Cav‐1 deficiency significantly aggravated lung structural remodelling and silicotic nodules formation in silicosis (Figure 2A). After 28 days of silica treatment, the *Cav*‐*1* ^−/−^ mice displayed more severe morphological damage, evidenced by more alveoli fusion, fibroblasts, collagen in the fibrotic walls (Figure 2A[c,d]), and more confluent silicotic nodules than the WT mice (Figure 2A[a,b]). The higher protein content in BALF further indicated that Cav‐1 deficiency aggravated lung injury after silica treatment (Figure S1). Based on Masson staining and IHC staining of collagen Ⅰ, Cav‐1 deficiency aggravated diffuse fibrosis in silicosis (Figure 2B,E), further confirmed in the WB analysis of collagen Ⅰ (Figure 2F). The silicosis severity increased in the lungs of silicotic *Cav*‐*1* ^−/−^ group than the silicotic nodules score (*p* < 0.05) and Ashcroft score (3.53 ± 0.81 vs. 5.29 ± 0.99, *p* < 0.05) of silicotic WT mice (Figure 2C,D).

### 3.3 Caveolin‐1 deficiency exacerbated silica‐induced inflammation

**FIGURE 3:**
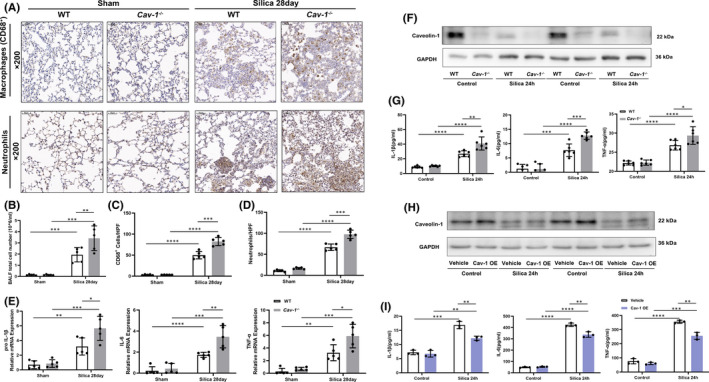
Caveolin‐1 (Cav‐1) deficiency exacerbated silica‐induced inflammation. (A) Immunochemistry staining of CD68 and myeloperoxidase (MPO) in the lung sections from wild‐type (WT) and *Cav*‐*1* ^−/−^ mice in the sham and silica 28 day groups. (B) The numbers of total cells in bronchoalveolar lavage fluid (BALF) from WT and *Cav*‐*1* ^−/−^ mice in the sham and silica 28 day groups (*n* = 5). (C, D) Quantification of total macrophages (CD68‐positive cells) and neutrophils (MPO‐positive cells) in lung parenchyma of WT and *Cav*‐*1* ^−/−^ mice in the sham and silica 28 day groups (*n* = 5). Relative mRNA levels of pro‐interleukin (IL)‐1β, IL‐6 and tumour necrosis factor‐ α (TNF‐α) in lung tissues from WT and *Cav*‐*1* ^−/−^ mice in the sham and silica 28 day groups (*n* = 5). Western blot analysis of caveolin‐1 of WT and *Cav*‐*1* ^−/−^ peritoneal‐derived macrophages (PDMs) after 24 h of silica exposure. Enzyme linked immunosorbent assay (ELISA) analysis of IL‐1β, TNF‐α and IL‐6 (*n* = 6) of WT and *Cav*‐*1* ^−/−^ PDMs after 24 h of silica exposure. (H–I) PDMs were transfected with the Cav‐1 plasmid (Cav‐1 OE) and empty plasmid (Vehicle). Western blot analysis of Cav‐1 and ELISA analysis of IL‐1β, TNF‐α and IL‐6 (*n* = 3) of PDMs in Cav‐1 OE and vehicle groups after 24 h of silica treatment. *p < 0.05, **p < 0.01, ***p < 0.001, ****p < 0.0001

CD68 and MPO are the most commonly used markers of macrophage and neutrophil.^22^ The IHC detected the accumulation of inflammatory cells in the lungs from WT and *Cav*‐*1* ^−/−^ silicotic mice (Figure 3A). The silicotic *Cav*‐*1* ^−/−^ mice presented greater neutrophil and macrophage numbers than the silicotic WT mice (macrophage: 49.88 ± 8.83 vs. 82.44 ± 9.71, *p* < 0.001; neutrophils: 66.84 ± 7.70 vs. 97.64 ± 9.92, *p* < 0.001). The total cell counts in BALF (1.94 ± 0.64 vs. 3.414 ± 1.11, *p* < 0.01) further verified the difference (Figure 3B–D). Cav‐1 deficiency enhanced the levels of pro‐inflammatory factors (pro‐IL‐1β, IL‐6 and TNF‐α) after silica treatment (*p* < 0.05) (Figure 3E). These results indicate that the Cav‐1 loss exacerbated pulmonary inflammation in silicosis. In vitro, the PDMs from WT and *Cav*‐*1* ^−/−^ mice mimicked the inflammation response of macrophages to silica exposure. Silica exposure reduced Cav‐1 levels in PDMs (Figure 3F). ELISA analyses showed that Cav‐1 deficiency enhanced the levels of pro‐inflammatory factors in silica‐treated PDMs (*p* < 0.05) (Figure 3G). Additionally, Cav‐1 over expression inhibited the secretion of pro‐inflammatory factors (*p* < 0.01) (Figure 3H,I). These results indicate that Cav‐1 negatively regulates silica‐induced inflammation in PDMs.

### 3.4 The possible mechanisms of Caveolin‐1 regulating silica‐induced inflammation

**FIGURE 4:**
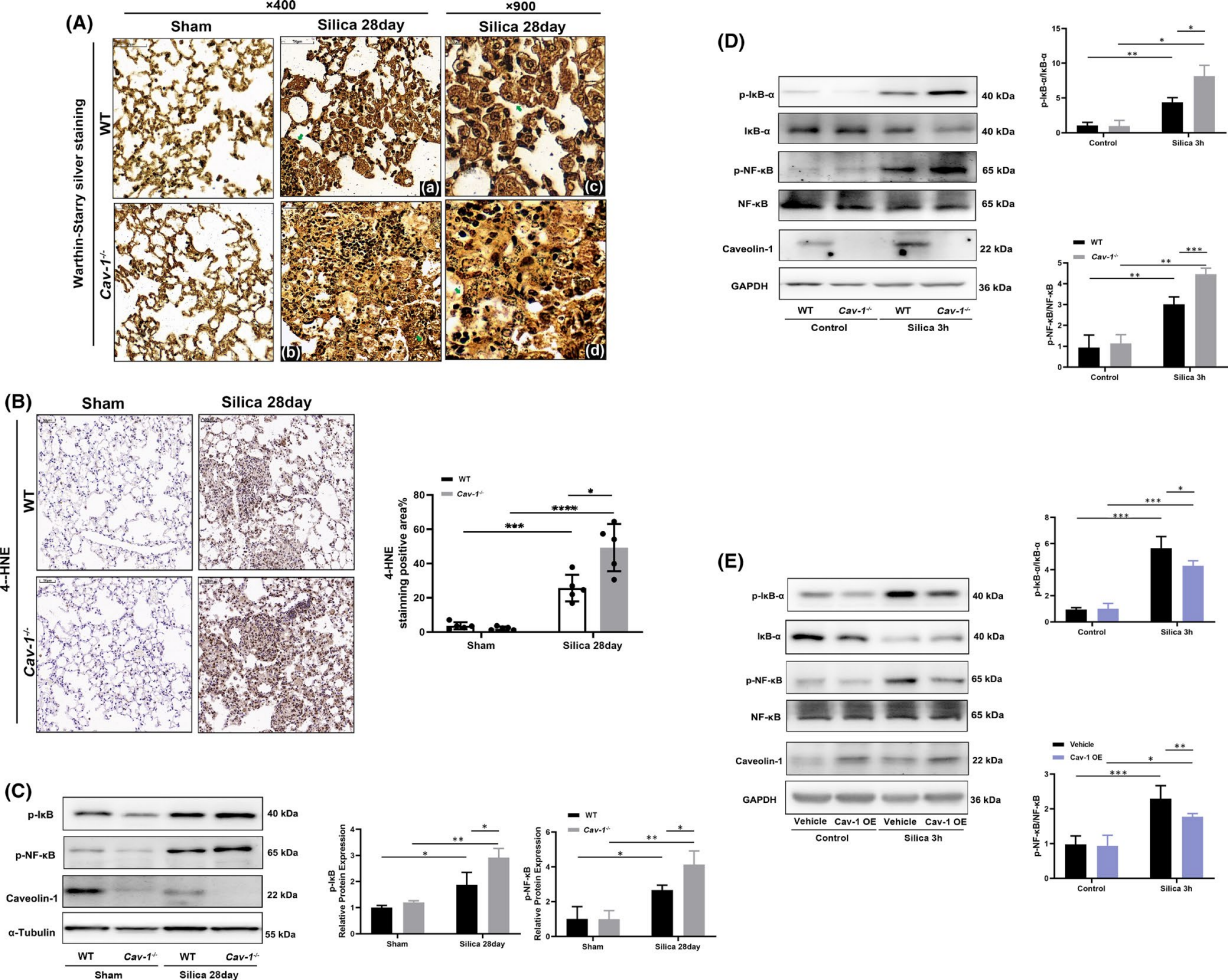
Possible mechanisms of Caveolin‐1 (Cav‐1) regulating silica‐induced inflammation. (A) Warthin‐Starry (W‐S) silver staining of lung sections from wild‐type (WT) and *Cav*‐*1* ^−/−^ mice in the sham and silica 28 day groups. W‐S silver staining shows the nuclear (a) and fibre (b), and much more silica dust deposited in macrophages in the lungs from *Cav*‐*1* ^−/−^ silicotic mice than the WT group (c, d). (B) Immunochemistry staining of 4‐hydroxynonenal (4‐HNE) in the lung sections from WT and *Cav*‐*1* ^−/−^ mice in the sham and silica 28 day groups. (C) Western blot analysis of the NF‐κB pathway in lung tissues from WT and *Cav*‐*1* ^−/−^ silicotic mice (*n* = 3). (D) Western blot analysis of the NF‐κB pathway in WT and *Cav*‐*1* ^−/−^ peritoneal‐derived macrophages (PDMs) after 3 h of silica exposure (*n* = 3). (E) PDMs transfected with the Cav‐1 (Cav‐1 OE) and empty (Vehicle) plasmids. Western blot analysis of the NF‐κB pathway in Cav‐1 OE and vehicle 3h after treatment (*n* = 3). *p < 0.05, **p < 0.01, ***p < 0.001, ****p < 0.0001

The sections from WT and *Cav*‐*1* ^−/−^ silicotic mice were treated with Warthin‐Starry (W‐S) silver staining to display the distribution of dust particles. Macrophages in the lungs from *Cav*‐*1* ^−/−^ silicotic mice had much more silica dust deposits than WT mice (Figure 4A[c,d]), indicating that Cav‐1 absence caused more silica dust accumulation in the lung tissue, thus causing continuous stimulation.

Silica induces pulmonary inflammation through oxidative stress.^23^, ^24^ The IHC of 4‐HNE shows that the *Cav*‐*1* ^−/−^ oxidative stress level is significantly higher than the WT, indicating that the same dose of silica dust causes more severe oxidative damage to cells due to the Cav‐1 absence (Figure 4B).

Moreover, the NF‐κB pathway is among the classic downstream pathways of oxidative stress. The NF‐κB signalling pathway was activated in lungs from silicotic mice, shown by upregulated levels of the NF‐κB subunit phospho‐p65, phospho‐IκB‐a, IκB‐a degradation. Cav‐1 deficiency promoted the activation of NF‐κB pathway in silicotic mice (*p* < 0.05) (Figure 4C) and PDMs treated with silica for 3 h (*p* < 0.05) (Figure 4D). In contrast, Cav‐1 overexpression downregulated the activation of NF‐κB signalling pathway (*p* < 0.05) (Figure 4E).

## 4 DISCUSSIONS

It was firstly found in this study that Cav‐1 levels were significantly reduced in the lung from silicosis patients and silicotic mice. Cav‐1 deficiency exacerbated the silica‐induced lung injury. Cav‐1 negatively regulates silica‐induced inflammation, in part, by regulating the NF‐κB pathway, silica intake and oxidative stress. Therefore, Cav‐1 has an essential protective effect in silicosis.

Cav‐1 is highly expressed in lung tissue, an anti‐inflammatory and anti‐fibrosis against lung injury.^10^, ^25^ This study suggests reduced Cav‐1 involved in the silicosis progression. Although the mechanism of silicosis is not fully elucidated, the belief is that silica‐induced macrophage inflammation induces initial injury and the insoluble silica causes continuous damage and inflammation, eventually leading to progressive fibrosis. Thus, the key treatment involves blocking the silica‐induced inflammation reaction. The in vivo and in vitro studies showed that silica‐induced inflammation significantly increased under Cav‐1 deficiency, and Cav‐1 overexpression inhibited the secretion of inflammatory factors. These results show that Cav‐1 is vital for regulating silicosis inflammation. However, the mechanism of silica‐induced inflammation is complex. The current belief is that direct silica toxicity induces oxidative stress,^26^ receptor recognition,^21^ phagocytic overload,^27^ thereby inducing inflammation. This study employed the W‐S silver staining to observe silica intake and IHC of 4‐HNE to observe the oxidative stress. The Cav‐1 absence triggered excessive silica deposition in cells, causing a stronger oxidative stress response. The NF‐κB pathway is important in oxidative stress‐induced inflammation. Systemic NF‐κB inhibition effectively improves lung tissue damage in silicosis mice.^28^ In vivo and in vitro experiments established that Cav‐1 negatively regulates NF‐κB pathway in silicosis.

These results suggest that decreasing Cav‐1 influenced the pathogenesis of silicosis and that enhancing Cav‐1 is a new candidate silicosis therapeutic strategy. In previous studies, three methods upregulated the Cav‐1 levels in various diseases. First, anti‐pulmonary fibrosis drugs (pirfenidone, fluorofenidone and ginsenoside Rg1) attenuated lung injury in bleomycin‐instilled animal models, secondary to enhancing caveolin‐1 expression.^29^, ^30^, ^31^ Secondly, knocking out the Cav‐1 E3 ubiquitin ligase, ZNRF1could reduce pulmonary inflammatory infiltration in LPS‐induced acute lung injury mice.^32^ Lastly, exogenous Cav‐1 peptide injection effectively upregulated Cav‐1 levels, therapeutically influenced myocardial remodelling, BLM‐induced pulmonary fibrosis and LPS‐induced inflammation.^33^, ^34^ Indeed, a Cav‐1‐derived peptide LTI‐03 entered phase 1 clinical trial with adaptation disease for idiopathic pulmonary fibrosis indication (NCT04233814). These studies suggest Cav‐1 as a potential therapeutic target for silicosis. This research team plans to exogenously inject Cav‐1 peptide to upregulate Cav‐1 expression in WT and *Cav*‐*1* ^−/−^ silicotic mice to clarify the Cav‐1 therapeutic effect on silicotic mice by observing injury, fibrosis and inflammation. Work is underway to explore the appropriate route and dose of the caveolin‐1 scaffolding domain peptide.

## 5 CONCLUSION

Cav‐1 significantly reduced in the lungs of silicosis patients and silicotic mice. Cav‐1 deficiency exacerbated silica‐induced lung injury and fibrosis. Cav‐1 negatively regulated silica‐induced inflammation, partly through NF‐κB pathway. Cav‐1 regulation is a potential treatment approach for silicosis.

## CONFLICT OF INTEREST

The authors have no conflicts of interest to declare.

## AUTHOR CONTRIBUTIONS

**Rongling He:** Conceptualization (lead); Investigation (lead); Writing – original draft (lead). **Xiangning Yuan:** Investigation (supporting). **Xin Lv:** Investigation (supporting). **Qingxiang Liu:** Resources (supporting). **Lijian Tao:** Writing – review and editing (supporting). **Jie Meng:** Writing – review and editing (lead).

## Supporting information

Fig S1

## Data Availability

The data that support the findings of this study are available upon request from the corresponding author.

## References

[1] Knight D , Ehrlich R , Fielding K , Jeffery H , Grant A , Churchyard G . Trends in silicosis prevalence and the healthy worker effect among gold miners in South Africa: a prevalence study with follow up of employment status. BMC Public Health. 2015;15:1258. 26686997 10.1186/s12889-015-2566-8 PMC4684919

[2] Franklin BS , Mangan MS , Latz E . Crystal formation in inflammation. Annu Rev Immunol. 2016;34:173‐202. 26772211 10.1146/annurev-immunol-041015-055539

[3] Barnes H , Goh NSL , Leong TL , Hoy R . Silica‐associated lung disease: an old‐world exposure in modern industries. Respirology. 2019;24(12):1165‐1175. 31517432 10.1111/resp.13695

[4] Hartert M , Senbaklavacin O , Gohrbandt B , Fischer BM , Buhl R , Vahld CF . Lung transplantation: a treatment option in end‐stage lung disease. Dtsch Arztebl Int. 2014;111(7):107‐116. 24622680 10.3238/arztebl.2014.0107 PMC3957052

[5] Chidlow JH , Jr., Sessa WC . Caveolae, caveolins, and cavins: complex control of cellular signalling and inflammation. Cardiovasc Res. 2010;86(2):219‐225. 20202978 10.1093/cvr/cvq075 PMC2856194

[6] Sanon VP , Sawaki D , Mjaatvedt CH , Jourdan‐Le SC . Myocardial tissue caveolae. Compr Physiol. 2015;5(2):871‐886. 25880516 10.1002/cphy.c140050

[7] Gvaramia D , Blaauboer ME , Hanemaaijer R , Everts V . Role of caveolin‐1 in fibrotic diseases. Matrix Biol. 2013;32(6):307‐315. 23583521 10.1016/j.matbio.2013.03.005

[8] Kulshrestha R , Singh H , Pandey A , Mehta A , Bhardwaj S , Jaggi AS . Caveolin‐1 as a critical component in the pathogenesis of lung fibrosis of different etiology: evidences and mechanisms. Exp Mol Pathol. 2019;111:104315. 31629729 10.1016/j.yexmp.2019.104315

[9] Cai L , Yi F , Dai Z , et al. Loss of caveolin‐1 and adiponectin induces severe inflammatory lung injury following LPS challenge through excessive oxidative/nitrative stress. Am J Physiol Lung Cell Mol Physiol. 2014;306(6):L566‐L573. 24441873 10.1152/ajplung.00182.2013 PMC3949082

[10] Liu J , Huang X , Hu S , He H , Meng Z . Dexmedetomidine attenuates lipopolysaccharide induced acute lung injury in rats by inhibition of caveolin‐1 downstream signaling. Biomed Pharmacother. 2019;118:109314. 31545263 10.1016/j.biopha.2019.109314

[11] Gao W , Shao R , Zhang X , Liu D , Liu Y , Fa X . Up‐regulation of caveolin‐1 by DJ‐1 attenuates rat pulmonary arterial hypertension by inhibiting TGFbeta/Smad signaling pathway. Exp Cell Res. 2017;361(1):192‐198. 29069575 10.1016/j.yexcr.2017.10.019

[12] Lei Y , Gao Y , Song M , Cao W , Sun X . Peroxynitrite is a novel risk factor and treatment target of glaucoma. Nitric Oxide. 2020;99:17‐24. 32222418 10.1016/j.niox.2020.03.006

[13] Fang Y , Zhang S , Li X , Jiang F , Ye Q , Ning W . Follistatin like‐1 aggravates silica‐induced mouse lung injury. Sci Rep. 2017;7(1):399. 28341862 10.1038/s41598-017-00478-0 PMC5428474

[14] Ohtsuka Y , Wang XT , Saito J , Ishida T , Munakata M . Genetic linkage analysis of pulmonary fibrotic response to silica in mice. Eur Respir J. 2006;28(5):1013‐1019. 16837500 10.1183/09031936.06.00132505

[15] Hubner R‐H , Gitter W , Eddine El Mokhtari N , et al. Standardized quantification of pulmonary fibrosis in histological samples. Biotechniques. 2008;44(4):507–517. 18476815 10.2144/000112729

[16] Wang X , Chen Y , Lv L , Chen J . Silencing CD36 gene expression results in the inhibition of latent‐TGF‐beta1 activation and suppression of silica‐induced lung fibrosis in the rat. Respir Res. 2009;10:36. 19439069 10.1186/1465-9921-10-36 PMC2698900

[17] Pineda‐Torra I , Gage M , De Juan A , Pello OM . Isolation, culture, and polarization of murine bone marrow‐derived and peritoneal macrophages. Methods Mol Biol. 2015;1339:101‐109. 26445783 10.1007/978-1-4939-2929-0_6

[18] Cassel SL , Eisenbarth SC , Iyer SS , et al. The Nalp3 inflammasome is essential for the development of silicosis. Proc Natl Acad Sci U S A. 2008;105(26):9035‐9040. 18577586 10.1073/pnas.0803933105 PMC2449360

[19] Lu M , Yang W , Peng Z , et al. Fluorofenidone inhibits macrophage IL‐1beta production by suppressing inflammasome activity. Int Immunopharmacol. 2015;27(1):148‐153. 25983199 10.1016/j.intimp.2015.05.008

[20] Zhang W , Zhang M , Wang Z , et al. Neogambogic acid prevents silica‐induced fibrosis via inhibition of high‐mobility group box 1 and MCP‐1‐induced protein 1. Toxicol Appl Pharmacol. 2016;309:129‐140. 27616297 10.1016/j.taap.2016.09.003

[21] Chan JYW , Tsui JCC , Law PTW , et al. Regulation of TLR4 in silica‐induced inflammation: an underlying mechanism of silicosis. Int J Med Sci. 2018;15(10):986‐991. 30013439 10.7150/ijms.24715 PMC6036162

[22] Zhou M , Tang W , Fu Y , et al. Progranulin protects against renal ischemia/reperfusion injury in mice. Kidney Int. 2015;87(5):918‐929. 25607110 10.1038/ki.2014.403

[23] Shukla A , Timblin CR , Hubbard AK , Bravman J , Mossman BT . Silica‐induced activation of c‐Jun‐NH2‐terminal amino kinases, protracted expression of the activator protein‐1 proto‐oncogene, fra‐1, and S‐phase alterations are mediated via oxidative stress. Cancer Res. 2001;61(5):1791‐1795. 11280724

[24] Lopes‐Pacheco M , Bandeira E , Morales MM . Cell‐based therapy for silicosis. Stem Cells Int. 2016;2016:1‐9. 10.1155/2016/5091838 PMC4811211 27066079

[25] Lin X , Barravecchia M , Matthew Kottmann R , Sime P , Dean DA . Caveolin‐1 gene therapy inhibits inflammasome activation to protect from bleomycin‐induced pulmonary fibrosis. Sci Rep. 2019;9(1):19643. 31873099 10.1038/s41598-019-55819-y PMC6928213

[26] Greenberg MI , Waksman J , Curtis J . Silicosis: a review. Dis Mon. 2007;53(8):394‐416. 17976433 10.1016/j.disamonth.2007.09.020

[27] Thibodeau MS , Giardina C , Knecht DA , Helble J , Hubbard AK . Silica‐induced apoptosis in mouse alveolar macrophages is initiated by lysosomal enzyme activity. Toxicol Sci. 2004;80(1):34‐48. 15056807 10.1093/toxsci/kfh121

[28] Di Giuseppe M , Gambelli F , Hoyle GW , et al. Systemic inhibition of NF‐kappaB activation protects from silicosis. PLoS One. 2009;4(5):e5689. 19479048 10.1371/journal.pone.0005689 PMC2682759

[29] Yu W , Guo F , Song X . Effects and mechanisms of pirfenidone, prednisone and acetylcysteine on pulmonary fibrosis in rat idiopathic pulmonary fibrosis models. Pharm Biol. 2017;55(1):450‐455. 27937011 10.1080/13880209.2016.1247879 PMC6130572

[30] Meng J , Zou Y , Hu C , et al. Fluorofenidone attenuates bleomycin‐induced pulmonary inflammation and fibrosis in mice via restoring caveolin 1 expression and inhibiting mitogen‐activated protein kinase signaling pathway. Shock. 2012;38(5):567‐573. 23042199 10.1097/SHK.0b013e31826fe992

[31] Zhan H , Huang F , Ma W , Zhao Z , Zhang H , Zhang C . Protective effect of ginsenoside Rg1 on bleomycin‐induced pulmonary fibrosis in rats: involvement of caveolin‐1 and TGF‐beta1 signal pathway. Biol Pharm Bull. 2016;39(8):1284‐1292. 27476938 10.1248/bpb.b16-00046

[32] Lee CY , Lai TY , Tsai MK , et al. The ubiquitin ligase ZNRF1 promotes caveolin‐1 ubiquitination and degradation to modulate inflammation. Nat Commun. 2017;8:15502. 28593998 10.1038/ncomms15502 PMC5472178

[33] Vogel ER , Manlove LJ , Kuipers I , et al. Caveolin‐1 scaffolding domain peptide prevents hyperoxia‐induced airway remodeling in a neonatal mouse model. Am J Physiol Lung Cell Mol Physiol. 2019;317(1):L99‐L108. 31042080 10.1152/ajplung.00111.2018 PMC6689745

[34] Shetty SK , Tiwari N , Marudamuthu AS , et al. p53 and miR‐34a feedback promotes lung epithelial injury and pulmonary fibrosis. Am J Pathol. 2017;187(5):1016‐1034. 28273432 10.1016/j.ajpath.2016.12.020 PMC5417006

