# Supplementary figures and images for: Caveolin‐1 negatively regulates inflammation and fibrosis in silicosis
Source: J Cell Mol Med. 2021 Dec 9;26(1):99–107. doi: 10.1111/jcmm.17045 (PMC8742238; doi:10.1111/jcmm.17045)

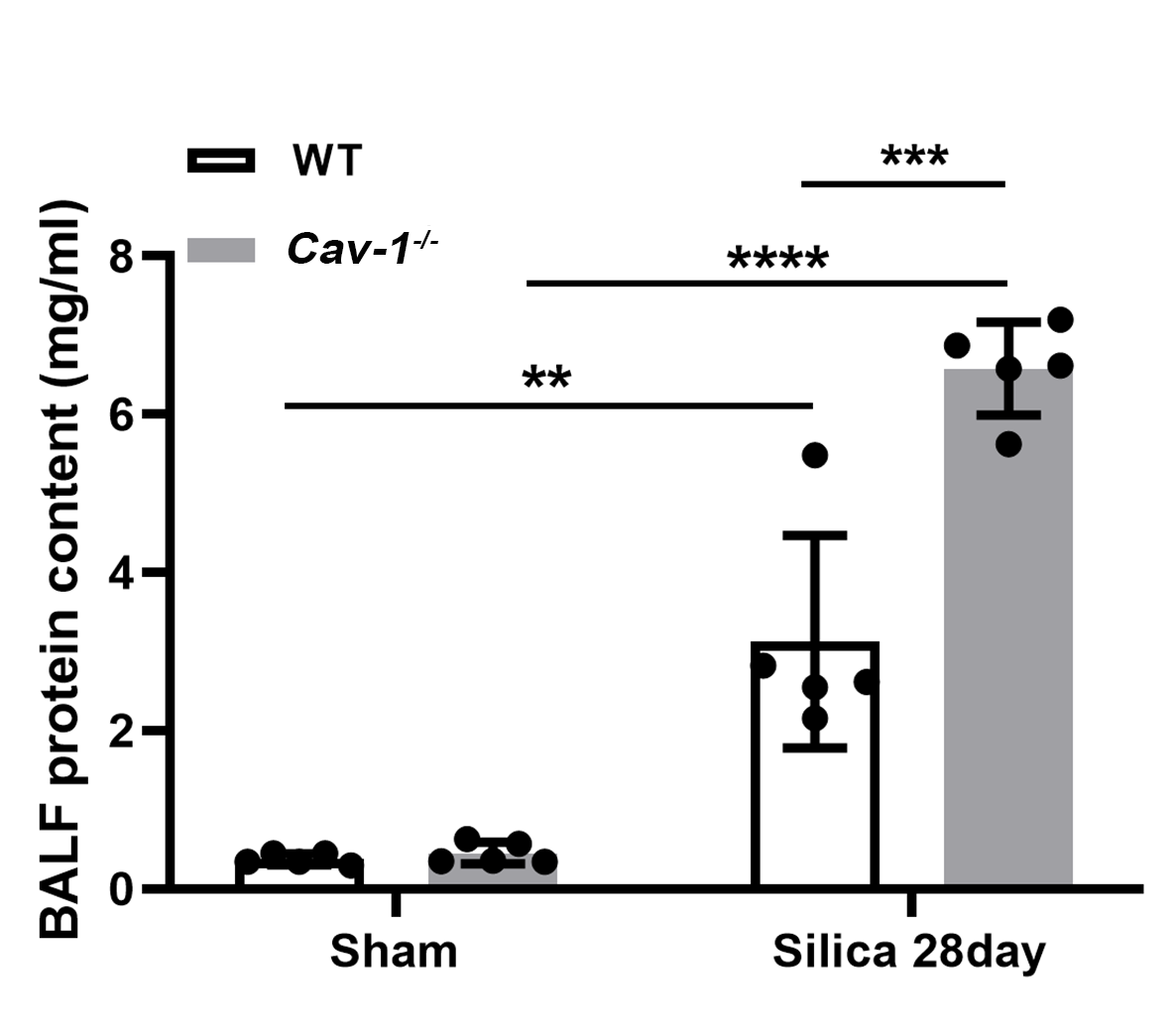

Supplement: Supplementary file 1 — Fig S1 [file JCMM-26-99-s001.tif]
